# A sensitive and simple targeted proteomics approach to quantify transcription factor and membrane proteins of the unfolded protein response pathway in glioblastoma cells

**DOI:** 10.1038/s41598-019-45237-5

**Published:** 2019-06-20

**Authors:** Chi D. L. Nguyen, Sebastian Malchow, Stefan Reich, Sascha Steltgens, Konstantin V. Shuvaev, Stefan Loroch, Christin Lorenz, Albert Sickmann, Christiane B. Knobbe-Thomsen, Björn Tews, Jan Medenbach, Robert Ahrends

**Affiliations:** 1 Leibniz-Institut für Analytische Wissenschaften-ISAS-e.V., 44227 Dortmund, Germany; 2 Schaller Research Group, University of Heidelberg and DKFZ, 69120 Heidelberg, Germany; 3 Molecular Mechanisms of Tumor Invasion, DKFZ, 69120 Heidelberg, Germany; 4 Translational Control Group, Biochemistry I, University of Regensburg, 93053 Regensburg, Germany; 5 Medizinische Fakultät, Ruhr-Universität Bochum, Bochum, 44801 Germany; 6 College of Physical Sciences, University of Aberdeen, Old Aberdeen, AB24 3UE UK; 7 Institute of Neuropathology, Medical Faculty, Heinrich-Heine-University Düsseldorf, 40225 Düsseldorf, Germany

**Keywords:** Proteomics, Cell signalling

## Abstract

Many cellular events are driven by changes in protein expression, measurable by mass spectrometry or antibody-based assays. However, using conventional technology, the analysis of transcription factor or membrane receptor expression is often limited by an insufficient sensitivity and specificity. To overcome this limitation, we have developed a high-resolution targeted proteomics strategy, which allows quantification down to the lower attomol range in a straightforward way without any prior enrichment or fractionation approaches. The method applies isotope-labeled peptide standards for quantification of the protein of interest. As proof of principle, we applied the improved workflow to proteins of the unfolded protein response (UPR), a signaling pathway of great clinical importance, and could for the first time detect and quantify all major UPR receptors, transducers and effectors that are not readily detectable via antibody-based-, SRM- or conventional PRM assays. As transcription and translation is central to the regulation of UPR, quantification and determination of protein copy numbers in the cell is important for our understanding of the signaling process as well as how pharmacologic modulation of these pathways impacts on the signaling. These questions can be answered using our newly established workflow as exemplified in an experiment using UPR perturbation in a glioblastoma cell lines.

## Introduction

The cellular concentrations of transcription factors (TFs) and transmembrane receptors (TMRs) are important parameters of their regulatory activity and potential. They determine cellular fate and the metabolic phenotype at multiple levels and are therefore key components in mathematical models describing the cellular decision-making^1^. However, many so far discovered signaling pathways are composed of low abundancy proteins (<2000 copy numbers per cell, approximately 10% of the current proteome)^2,3^ and only sparse information on concentrations of the involved TFs and TMRs are available^4^. Their quantitative analysis has been a challenge in proteomics^5,6^ hindering analyses of signaling protein expression levels. To address this limitation, proteomics experiments usually employ either exhaustive subcellular fractionation, multidimensional separation or enrichment of genetically tagged proteins or peptides using antibodies^7–10^. However, multi-step enrichment strategies are laborious, time consuming and prone to biases, limiting their application in biological context with multiple sample types and replicates. Consequently, semi-quantitative immunoblots are still used in the daily routine for estimating the levels of well-known TFs and other low abundancy proteins^11,12^. Targeted MS approaches offer the potential to overcome some of these challenges: once established they are relatively fast to perform and highly reproducible. In addition, the use of isotope-labeled peptides that serve as internal standards (IS) allows for absolute quantification^13^. Although targeted proteomics workflows such as Selected Reaction Monitoring (SRM) are in principle highly sensitive, they often suffer from signal interferences at the MS/MS level, limiting their broad application^14^, particularly for very low abundancy proteins in highly complex mixtures. Since its first introduction by Peterson *et al*. and Gallien *et al*. in 2012 on an orbitrap instrument^14,15^, parallel reaction monitoring (PRM) has been showing its advantages over the traditional SRM approach in term of sensitivity and selectivity^16^. In 2016, Bourmaud *et al*. introduced different types of acquisition methods in PRM, where one can increase the injection time of the trapping device to enhance the sensitivity or the orbitrap resolution for better selectivity^17^. In the current study, we combined the long injection time and high resolution properties as well as the isotope-labeled peptides to enhance both the sensitivity and selectivity for a straightforward quantitative analysis of low abundancy signaling proteins. For the development and application of the targeted proteomics approach, we chose as a model system the unfolded protein response (UPR). UPR signaling involves a variety of low abundancy TFs and TMRs^18^ and plays a critically important role in various disorders^18,19^.

The UPR pathway is triggered by the accumulation of unfolded proteins in the endoplasmic reticulum (ER) and aims to re-instate cellular proteostasis, or, otherwise to trigger apoptosis^20,21^. In general, the UPR utilizes three main branches to sense perturbations to ER homeostasis and to initiate adaptive changes to gene expression, mRNA- and protein turnover^20,22–24^. It has been shown to for example in the context of cancer, to contribute to angiogenesis, sustained proliferation, and resistance to pharmacological treatment^25–27^. Given its broad clinical impact, the UPR has emerged as an attractive target for therapeutic intervention^28,29^.

Although quantitative proteomic studies have been performed to investigate the UPR, proteins central to this signal transduction pathway (such as PERK, ATF6, IRE1a, CHOP, XBP1, GADD34 and ATF4) proved as notoriously difficult to detect and to quantify^3,30–32^. Here we present a high resolution targeted proteomics workflow with enhanced sensitivity and selectivity for the analysis of low abundancy proteins that does not require enrichment, subcellular fractionation or two-dimensional separation steps. We highlight the critical instrument parameters of a quadrupole-Orbitrap mass spectrometer needed to increase the sensitivity by at least one order of magnitude allowing the quantification of TFs and TMR down to the low attomol range. We applied this workflow to assess the dynamics and copy numbers of the major signaling proteins of the UPR before and after its induction in the glioblastoma cell line LN-308.

## Results

### A one-stop workflow for targeted proteomics of low abundancy protein by PRM

**Figure 1:**
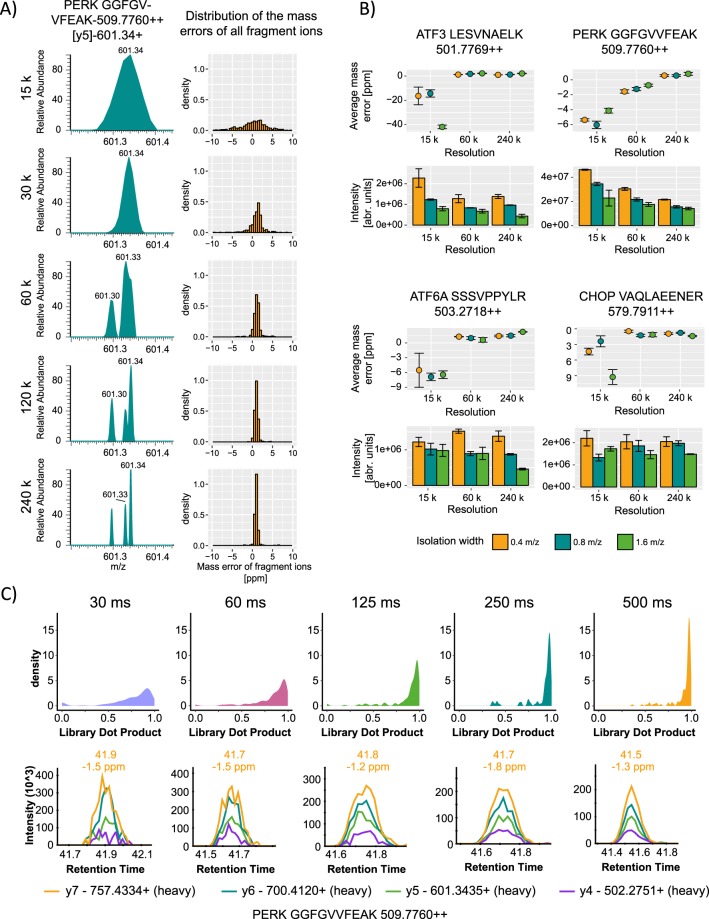
Establishing a high resolution (HR) targeted proteomics approach for low abundancy proteins. (**A**) Effect of different resolution on the mass separation and mass deviation. Right panel displays the resolution effect on a representative example of the PERK peptide GGFGVVFEAK transition y5, left panel shows the distribution of the average mass deviation at different resolution for all analyzed precursors. (**B**) Effect of different isolation widths on the mass accuracy and intensity at three different resolutions 15 k, 60 k and 240 k: Isolation width was set to m/z values of 0.4, 0.8 and 1.6, injection time to 500 ms and an AGC target value of 3 × 10^6^ was used for all measurements respectively. (**C**) Global effect of the injection time on the dot product at high resolution (upper panel), below representative example of injection time effects on ion traces of the PERK peptide GGFGVVFEAK at high resolution (240 k). All measurements were repeated at least 3 times.

In order to develop a quantification method for low abundancy proteins with high specificity, we started with the previously described MS acquisition method called parallel reaction monitoring PRM^17^. In brief, separated peptide precursors are isolated based on their mass to charge ratio, fragmented and all fragments are analyzed in parallel in an orbitrap mass analyzer. PRM methods have the potential of high selectivity by increasing the resolution and therefore the capability to distinguish between target ions and matrix, making it superior compared to conventional SRM techniques. Using an in-silico spectral library, pH8 reversed phase sub-fractionation and a PRM screen on selected targets, we identified the peptides of interest central to the key UPR proteins (Supplementary Fig. S1). Then, we optimized the PRM workflow to its highest level of sensitivity and selectivity to detect all central UPR proteins in one simple and straightforward measurement. As we used the conventional PRM settings for targeted analysis of proteins: 60,000 resolution, 80 ms injection time, isolation width m/z value of 1.6 and 3 × 10^6^ AGC target value, we could not detect the majority of the proteins involved in the UPR (Supplementary Fig. S2A). However, by increasing the resolution to 240,000, allowed the detection of multiple so far undetectable peptide signals. We also observed that if the resolution is set below 120,000 the likelihood of integrating multiple false fragment ions is high and the mass error increases above 5 ppm (Fig. 1A) leading to an elevated chemical noise during the ion extraction and integration. Especially for low abundancy proteins, this property plays an important role to distinguish between the background and ions of interest.

We tested further different isolation widths at three different resolution powers (15,000; 60,000; 240,000) while keeping the injection time at 500 ms. We observed that the majority of the monitored precursor ions at isolation width m/z value of 0.4 had increased signal to noise ratio (S/N) compared to m/z values of 0.8 and 1.6 (Figs 1B, S2B). Furthermore, the isolation width has much less impact on the mass accuracy of the precursor ions compared to the orbitrap resolution (Figs 1B, S3)

To enhance the sensitivity, we then increased the injection time stepwise from 30 ms to 500 ms while keeping the resolution at 240,000 and isolation width m/z value of 0.4. We compared the dot product (dopt) of all the acquired precursor ions (heavy and light) from all the replicate measurements to a library spectrum. The library spectrum was obtained using 2 µg of treated samples, 59–980.8 amol of IS per injection and acquired at 240,000 resolution, isolation width m/z value of 0.4 and 500 ms of injection time. The dot product is a measure of similarity between the measured spectrum and the library spectrum in our case^33^. It ranges from 0.00 for no similarity to 1.00 for absolute similarity. At an injection time of 500 ms individual fragment ion signals as well as the ratios between these were stabilized (more dot products reached 1) allowing a more robust quantification with improved S/N (Fig. 1C).

**Figure 2:**
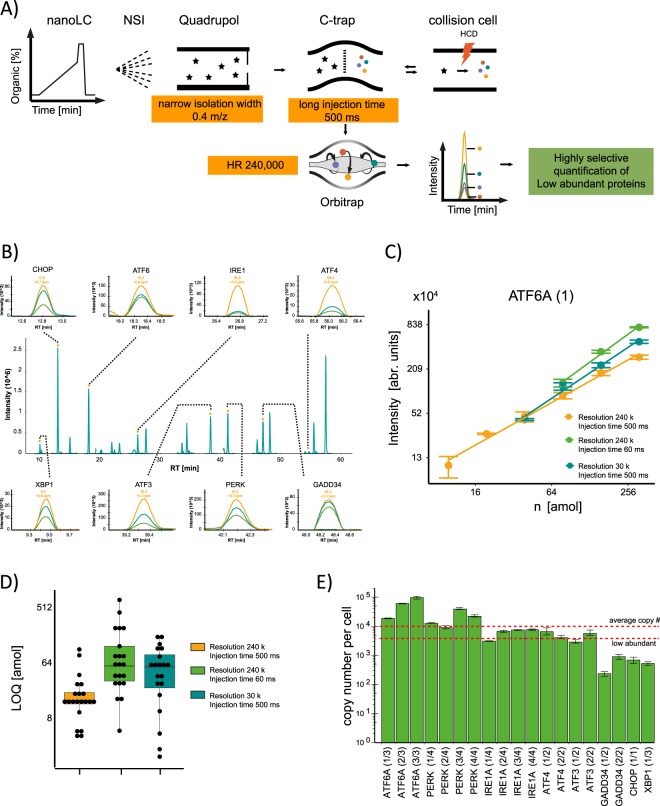
Application of the HR targeted workflow for the quantification of UPR proteins. (**A**) Final setup and instrument parameter used for quantification. (**B**) Representative PRM traces of the targeted peptides corresponding to UPR proteins and their fragment ion traces. (**C**) Calibration curves for an ATF6 assay with different parameter set-ups. Both x and y axis are log 2 scaled (**D**) Limit of quantification is lowered by high resolution and longer injection times. (**E**) Measured copy numbers per cell of the analyzed UPR proteins in the glioblastoma cell line LN-308. The y-axis is presented in log10 scale. All measurements were repeated at least 3 times. To calculate the copy numbers/cell, 3 biological replicates were used and the error is presented as standard deviation.

Combining the high resolution of 240,000, a narrow isolation width m/z value of 0.4 and a high injection time of 500 ms, allowed us to detect and quantify all low abundancy UPR signaling proteins in a single experiment using 1–2 µg of sample. Compared to the conventional PRM and the SRM approaches, the scan rate of this method is low due to the long injection time and scan time, which in turn leads to limited data points over the peak and lower multiplexing ability. To overcome these limitations we optimized the workflow and fixed the scheduled retention time window to 2 min and select peptides which elute relatively separated from each other (see Peptide Selection part in Methods and Fig. 2B).

We were able to detect all the UPR proteins in LN-308 glioblastoma cells as well as in Hek293 cells, in Hela cells and in a human primary fibroblasts cell line at basal level. The extracted ion chromatograms of all measurements can be found in this Skyline Panorama link (https://panoramaweb.org/signaling_targeted_proteomics_2018.url)

### Implementation of the optimized PRM workflow to quantify low abundancy UPR proteins at basal level

To determine the concentrations of the major proteins involved in the UPR and to monitor their steady state levels after ER stress induction, we applied the optimized PRM workflow to the cell line LN-308. Using high resolution, high injection time and a narrow precursor isolation width (Fig. 2A) we quantified the major UPR signaling proteins (represented by 21 peptides): IRE1a, PERK, ATF6A, GADD34, ATF4, ATF3, CHOP and XBP1 (Fig. 2B, Supplementary Table S1).

Calibration curves for all peptides (heavy) were analyzed using three parameter sets: 240 k-500 ms; 240 k-60 ms; 30 k-500 ms (Figs 2C, S4). The starting amount of the dilution series ranged from 0.5–7.7 amol to 59–980.8 amol on column depending on the MS response of each individual peptide (Supplementary Table S2). The high resolution and high injection time parameter set (240 k, 500 ms) increased the dynamic range of detection for all the proteins leading to an overall better limit of quantification underscoring beneficial use of the here established workflow (Fig. 2D). For the targeted proteins LOQs ranging from 4 amol (GADD34, APLSPSLLIR) to 103 amol (ATF6A, AEPQPLSPASSSYSVSSPR) were achieved (Supplementary Table S1).

Using calibration curves for each individual peptide, we determined first the absolute amount and then the cellular copy numbers of the proteins central to the UPR which ranged from ~400 for XBP1 to ~60,000 for ATF6 (Fig. 2E and Supplementary Table S3). On average, the copy number per cell across all proteins was rather low (~12,000), demonstrating that the UPR pathway mostly involves low abundancy proteins which explains the experimental challenges to quantify the UPR. Compared to comprehensive proteomics methods under stress conditions^3,30,32^ our simplified workflow now allows rapid analysis of all UPR key players (including the low abundancy receptors and effectors) enabling us to quantitatively assess this clinically important signaling pathway under various conditions.

### Quantification of major signaling proteins upon induction of the UPR

Upon accumulation of unfolded proteins in the ER, immediate signaling through the three UPR receptors (IRE1, PERK and ATF6) is triggered. Their activation is driven by post-translational modification (phosphorylation of IRE1 and PERK) or processing (proteolytic cleavage after translocation into the Golgi of ATF6). Activation of PERK results in a rapid attenuation of translation and hence reduced protein loading into the ER^34^. In parallel, adaptive transcriptional changes are induced by the major UPR transcription factors ATF6, XBP1 and ATF4^18^. Production of XBP1 and ATF4 is controlled by IRE1 and PERK signaling, respectively.

While the initial phase of the UPR (activation of IRE1, PERK, and ATF6) does not per se require changes of protein abundance of the involved regulatory factors, the later phases of the UPR impact on gene expression and result in adaptive proteomic changes to allow the system to settle into a new equilibrium. This is further facilitated by the induction of negative feedback loops that dampen the response to prevent an overshooting response (such as e.g. induction of GADD34, a regulatory subunit of the protein phosphatase 1 to counteract PERK signaling)^35^.

However, the UPR-driven, dynamic regulation of UPR-effectors has not yet been quantitatively addressed. Moreover, upon triggering of the UPR, a change in the steady state level of UPR key factors was reported^36,37^, suggesting that adaptation to ER stress also affects UPR signaling through changes in the abundance of signaling factors.

To gain further insight into the dynamic regulation of the abundance of UPR proteins, we applied the optimized PRM workflow after chemical induction of ER stress in the glioblastoma cell line LN-308. For this, we employed either the N-glycosylation inhibitor Tunicamycin (TM, 2.5 µg/ml) or Thapsigargin (TH, 200 nM), an inhibitor of the sarco/endoplasmic reticulum (ER) Ca^2+−^ATPase (SERCA)^16^. We treated the cells for different time periods and directly harvested the cells.

**Figure 3:**
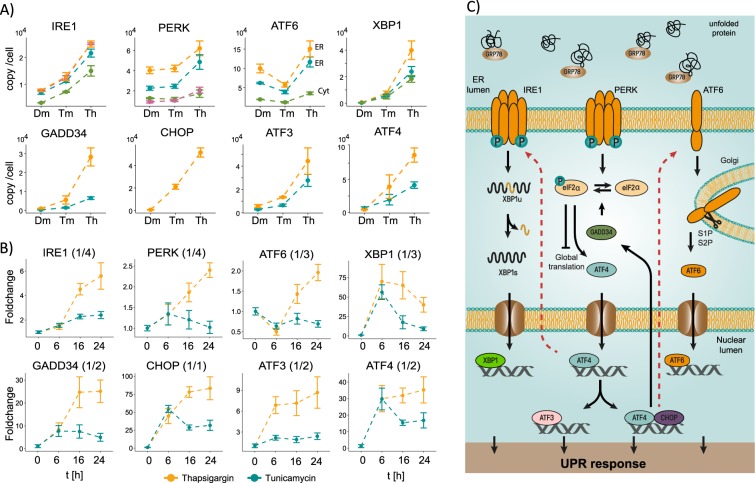
Quantification of the UPR signaling pathway upon UPR activation. (**A**) Comparison of different UPR protein derived peptides in non-stressed cells and after 24 hr with induction of the UPR by the inhibition of N-Glycosylation (Tunicamycin, 2.5 µg/ml) or disruption of the Ca^2+^ homeostasis (Thapsigargin, 200 nM). (**B**) Time curves of the UPR over a duration of 6, 16 and 24 hr after Tunicamycin and Thapsigargin treatment. All measurements were performed with 3 biological replicates and the error is presented as standard deviation. (**C**) Schematic representation of UPR signaling as described in the text. The dashed red line indicates feedback on the abundance of the receptor proteins that reside in the ER membrane.

In general, we detected abundance changes for all UPR signaling proteins after 6, 16 and 24 hr of incubation (Fig. 3A,B). However, the responses between the TM and TH treatment are different in strength and duration. For IRE1a, PERK, ATF4, GADD34, CHOP and XBP1, the inhibition of N-glycosylation leads to an increase in copy numbers of approximately 5-fold whereas the inhibition of SERCA leads to an approximately 17-fold upregulation. Moreover, while TH treatment fosters a sustained increase in the UPR-related factors, attenuation of the cellular response is observed 16 hr after inhibition of N-glycosylation by TM and the levels of many UPR proteins are reduced (Fig. 3B). For the different proteins we detected different response curves. Proteins such as XBP1s, ATF4, and ATF3 are induced early followed by GADD34 and CHOP, while the induction of IRE1a, PERK and ATF6 is delayed. This indicates an additional level of feedback regulation to adapt the receptor concentration level to sustain UPR signaling during ongoing ER stress (Fig. 3C).

### Quantification of the UPR proteins in different glioblastoma cell lines

**Figure 4:**
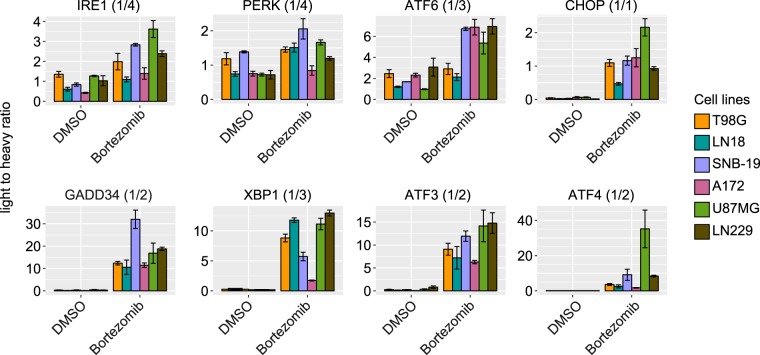
Quantification of UPR proteins in different glioblastoma cell lines non-treated and treated with Bortezomib using Orbitrap Fusion Lumos Tribrid Mass Spectrometer. Applying the optimized PRM assay, the expression level of the low abundancy UPR proteins at basal level and under treatment with 1 nM Bortezomib could be determined. The ratios between internal standard peptide and the endogenous peptide (light to heavy ratio) are presented. All the measurements were performed with three biological replicates.

To validate the usefulness of our newly established workflow and to extend our data, we investigated the expression levels of the UPR proteins in 6 different glioblastoma cell lines at basal level and under treatment with 1 nM Bortezomib for 24 hr^15^. Bortezomib (Velcade, Millennium Pharmaceuticals, Inc., Cambridge, MA) is a proteasome inhibitor, which is used in the clinic for the treatment of multiple myeloma and mantle cell lymphoma^15,38^. Inhibition of the proteasome results in accumulation of defective proteins in the cell especially in ER leads to ER stress and degradation of anti-apoptotic proteins, which then results in programmed cell death of tumor cells. However, resistance against Bortezomib has been observed in many types of tumors^39^. The aggressiveness of certain tumor types has been linked to the activity of the UPR as tumor cells can facilitate the survival events to overcome harsh conditions caused by therapeutic drugs^40,41^. To further investigate the role of the UPR in glioblastoma cell lines, we applied the optimized PRM approach to quantify the UPR protein expression levels in 6 different glioblastoma cell lines, namely A172, U87MG, LN18, LN229, SNB-19 and T98G under basal condition and after treatment with 1 nM Bortezomib for 24 hr (Fig. 4). Furthermore, we transferred the optimized PRM assay on a newer generation of Orbitrap instrument (Orbitrap Fusion Lumos Tribrid Mass Spectrometer) with more effective parallelization time resulting in a higher scan rate and less fluctuation of scan frequency (Supplementary Fig. S5). Using the Bortezomib treatment and different cell lines we observed diversity in expression levels of UPR proteins at basal condition as well as after treatment with Bortezomib. Overall, the elevated UPR activation could be observed in all 6 cell lines and in all 3 UPR branches (Fig. 4). However, the level of activation was different between cell lines. As expected, the upregulation of UPR receptor proteins (ATF6, PERK and IRE1) is less pronounced compared to the effector proteins (ATF3, ATF4, XBP1, GADD34 and CHOP).

## Discussion

One of the major challenges in proteomics is the detection of low abundancy proteins. Among these low abundancy proteins are important signaling molecules such as transmembrane receptors and transcription factors. So far many of these proteins can only be analyzed semi-quantitatively using for example Western blotting techniques which in turn are dependent on the availability of high quality antibodies or nanobodies. From the proteomics perspective these questions have been addressed using numerous different subcellular and 2D- fractionation techniques as well as biochemical enrichment and separation approaches^30,31^. Here, we optimized a conventional PRM targeted proteomics workflow to its highest level of sensitivity and selectivity, which enable the detection and quantification of low abundancy proteins in one simple and straight forward measurement. Applying the optimized PRM workflow, the amount of measurement and sample preparation time is drastically reduced since extensive fractionation or enrichment approaches prior to analysis are no longer required. However, only a limited number of proteins or peptides can be covered per assay due to the low scan rate, which can be overcome by selection of peptides based on their elution profile and/or application of the assay on a faster orbitrap instrument such as the Orbitrap Fusion Lumos Tribrid Mass Spectrometer.

As a proof of concept we analyzed all key components of the UPR using our newly established workflow. The UPR plays an important role in many different diseases and affects major pathways that contribute to cancer progression and prognosis, including angiogenesis, metastasis, genome stability, inflammation and drug resistance^42^. Its quantitative analysis is therefore of great clinical and prognostic importance. Using our improved methodology, we could for the first time detect and quantify all key proteins of the UPR in a single and straightforward analysis.

In this study, the targeted high resolution proteomics approach allowed not only to identify the major regulators of the UPR signaling pathway, but also to quantify their cellular abundance in non-stressed cells and after triggering of the UPR. These factors span concentration levels from hundreds to 60,000 molecules per cell and their abundance dynamically changes upon ER stress. Therefore, the UPR is not only regulated through signaling that involves posttranscriptional and posttranslational modification, but also driven by changes to the concentrations of proteins that play key roles in the UPR, including the receptors that reside at the ER membrane. The change in copy number of the UPR receptors - which occurs relatively late after the onset of ER stress - suggests an increase in the signaling capacity of the entire UPR network and indicates an adaptive feedback. The latter is supported by the finding that the UPR effector proteins CHOP and ATF4 can associate with the promoter region of ATF6 and that ATF4 can induce IRE1a expression upon UPR induction^37,43^. Moreover, this suggests extensive crosstalk and feedback between the different arms of the UPR.

ER stress-mediated signaling via PERK and IRE1a differs in several aspects from ATF6 signaling. Upon accumulation of unfolded proteins in the ER, PERK and IRE1a are activated by (auto)phosphorylation. Regulation of their downstream targets is based on catalytic turnover which results in an amplification of the signal (a single activated UPR receptor can process or phosphorylate a large number of target molecules). Finally, signaling through PERK and IRE1a can be attenuated by dephosphorylation, restoring the initial state of the system.

In contrast, upon ER stress ATF6 translocates into the Golgi where proteolytic cleavage liberates its N-terminus which then translocates to the nucleus and acts as a transcription factor. Once activated and processed by proteolytic cleavage, ATF6 cannot be recycled. In order to restore the initial state of the cell, the ATF6 fragments have to be removed by proteolysis and new molecules of ATF6 have to be synthesized. In line with this, our data show that the ATF6 protein abundance decreases after 6 hr of ER stress, but recovers at later time points (16 hr and 24 hr) (Fig. 3C). Strikingly, a very similar behavior is observed for peptides derived from the N-terminus of ATF6 that directly participates in transcriptional regulation, and peptides derived from the C-terminus that remains anchored in the membrane. Moreover, the ATF6 signaling pathway does not include a catalytic amplification loop and hence necessitates higher copy numbers of the protein per cell to achieve a comparable signaling strength. Therefore, it is not surprising that ATF6 is the most abundant UPR receptor (approx. 60,000 copies per cell), exceeding the levels of both PERK (approx. 20,000 copies per cell) and IRE1a (approx. 6,000 copies per cell) (Supplementary Table S3). Further, we performed the optimized PRM assay on six different glioblastoma cell lines at basal level and after treatment with Bortezomib, a therapeutic agent widely used in current chemotherapy cancer treatment. For the first time, we could demonstrate that sensors and effectors are controlled differentially depending on treatment and origin. Numerous studies show that the UPR works as a pro-oncogenic signaling network that can contribute to cancer progression and affect clinically important parameters^42^. In support of this, high levels of ATF6 or PERK have been associated with poor prognosis and reduced survival^44,45^. Unfortunately, most pre-clinical analysis tools still employ immuno-assays, and thus the analysis of entire signaling pathways is challenging. Moreover, these assays provide only limited quantitative information which are urgently needed if the potency of drug candidates such as MKC-3946, an IRE1a RNase inhibitor^46^ or other small molecule inhibitors of the UPR are going to be tested^47^. Our PRM assay presents an attractive solution, providing high detection sensitivity and quantitative data for prognostic analyses and efficacy testing.

Here we demonstrate a PRM method optimization, which has the potential for a broad applicability in proteomics. It is highly reproducible and flexible in its setup and as such adaptable to other pathways and samples. Our assay fulfills the requirement of Tier 2 measurement of the Guidelines for Targeted MS Manuscripts of Molecular and Cellular Proteomics journal (updated on 9^th^ of January 2018). Moreover, we believe that it can also be applied to signaling lipidomics^48^ and other fields of targeted MS approaches suffering from sensitivity issues^49^. With increasing MS performance speed, also the target molecule panel can very likely be expanded by a factor of 5 (covering about 100 molecules), which will then strengthen the employment of the mass spectrometry based analysis of signaling pathways as an alternative to conventional diagnostic tools.

## Methods

### Cell culture

LN-308 cells (RRID:CVCL_0394) were cultured in Dulbecco’s modified Eagle’s medium supplemented with 10% FBS, 100 units/ml penicillin and 100 µg/ml streptomycin at 37 °C and 5% CO_2_. For MS experiments, cells were seeded on the day prior to treatment, so that at the day of treatment the confluency reached 50%. ER stress was induced by addition of either 200 nM Thapsigargin (Sigma-Aldrich, Hamburg, Germany) or 2.5 µg/ml Tunicamycin (Sigma-Aldrich, Hamburg, Germany) to the medium and followed by incubation for 6, 16 and 24 hr. The concentration of Thapsigargin and Tunicamycin was adapted from Harding *et al*.^16^. As control, cells were incubated with DMSO for the same time periods. After incubation cells were washed with ice-cold PBS, scraped from the cell culture dishes and pelleted by centrifugation at 4 °C and 2500 rpm for 5 min in a benchtop centrifuge. Cell pellets were frozen in liquid N_2_ and stored at −80 °C until further use. For the experiment with six glioblastoma cell lines, they were cultured in Dulbecco’s modified Eagle’s medium supplemented with 10% FBS, 100 units/ml penicillin and 100 µg/ml streptomycin at 37 °C and 5% CO_2_. 1.75 × 10^6^ cells were seeded a day before treatment. The cells were treated with 1 nM Bortezomib^15^. As negative control, DMSO was used at 0.006%. After 24 hr of treatment, the cells were washed twice with ice cold PBS, collected by scrapping down and centrifugation (1500 rpm, 5 min, 4 °C). The cell pellets were then flashed frozen and stored at −80 °C for further use.

### Peptide selection

The peptides were selected based on the in-silico-digest of the protein of interest that they are unique on the proteome level and carry no miss cleavage or ragged end. In order to have enough data points over the peak, we restricted the number of concurrent precursors at any elution time to the maximum of 3 precursor pairs (heavy and light). Based on these criteria, we selected peptides that elute relatively well-separated from each other and we applied a scheduled PRM method with a 2 min of retention time window. For this we reduced the number of precursors from 100 for 9 proteins (IRE1, PERK, ATF6, ATF3, ATF4, CHOP, GADD34, XBP1 and eIF2A) to 21 peptides for 8 proteins (IRE1, PERK, ATF6, ATF3, ATF4, CHOP, GADD34 and XBP1).

### Synthesis of synthetic isotopic labelled (SIL) peptides

The IS peptides were synthesized in-house using a Fmoc-chemistry-based solid-phase peptide synthesis system (Syro II, MultiSynTech, Witten, Germany). After synthesis, the peptides were purified using reverse phase chromatography. The cysteine-containing peptides were carbamidomethylated using 10 mM DTT at 56 °C for 30 min and subsequently 30 mM IAA for 30 min at 25 °C in the dark. The purified peptides were quantified using amino acid analysis (AAA) approach consisting of a gas-phase acid hydrolysis and a fluorescence labelling step. The quantification was performed based on the fluorescence absorption signal on an Acquity UHPLC H class system (Waters, Milford, MA, USA). The peptides were solubilized in 30% ACN and 0.1% TFA and diluted in 0.1% TFA for measurement.

### Sample preparation

The cell pellets were lysed in 300 µl lysis buffer (1% SDS, 150 mM NaCl, 50 mM Tris-HCl (pH7.8), 1 × cOmplete (1 tablet per 50 ml), 1 µl Benzonase Nuclease (Merck KGaA, Darmstadt, Germany) and 1 µl of 1 M MgCl_2_ was added to each sample. After incubation at 37 °C for 30 min, the cell lysates were centrifuged for 30 min at 18.000 × g and 4 °C. The protein concentration of each sample was determined using a bicinchoninic acid (BCA, Thermo Scientific Hamburg Germany) assay. As starting material, 100 µg of total protein was used for sample cleanup and proteolytic digestion using a filter-aided sample preparation protocol (FASP). The samples were reduced with 10 mM Tris(2-carboxyethyl)phosphine hydrochloride (TCEP, Sigma-Aldrich, Hamburg, Germany) at 56 °C for 30 min and alkylated in the dark for 30 min with 30 mM Iodoacetamide (IAA, Sigma-Aldrich, Hamburg, Germany) at 25 °C. The cell lysates were diluted to less than 0.2% SDS with freshly prepared Urea buffer (8 M Urea in 100 mM Tris-HCl pH8.5) and loaded onto a centrifugal device (30 kDa molecular weight cut-off, Pall Laboratory, Washington, USA). Centrifugation occurred at 13,500 × g 25 °C. After sample loading, the filter membrane was washed three times with 100 µl Urea buffer to remove traces of SDS, followed by three times washing with 100 µl 50 mM NH_4_HCO_3_ (pH 7.8). For the enzymatic digestion, Trypsin was used at a ratio of 1:25 (w/w, protease to substrate) in 100 µl digestion buffer (0.2 M Guanidine-HCl, 2 mM CaCl_2_, 50 mM NH_4_HCO_3_ (pH7.8)). After the digestion (max. 15 hr at 37 °C), the tryptic peptides were collected by centrifugation with 200 µl of 25 mM NH_4_HCO_3_ (pH7.8) and acidified with 10 µl 10% trifluoroacetic acid (TFA). The digestion efficiency was checked by monolithic reverse phase separation as described in Burkhart *et al*.^50^. For the method development, 0.1 to 2 fmol of IS peptides per injection were spiked in. As matrix, we used 2 µg of tryptic digest from LN-308 cell non treated or treated with 200 nM Thapsigargin for 24 hr. For the calibration curves of three investigated combinations 240,000 resolution – 500 ms fill time, 240,000 resolution – 60 ms fill time and 30,000 resolution – 500 ms fill time, dilution series were performed started at concentrations from 59–980.8 amol/µl depending on the MS response of each individual peptide. After 7 dilution steps with a factor of 2, the final concentration varied from 0.5 to 7.7 amol/µl. The concentration of each peptide at each dilution step is given in the Supplementary Data (Supplementary Table S2). As matrix, we used 2 µg of tryptic digest of LN-308 cells treated with 200 nM Thapsigargin for 24 hr. For the quantification of the UPR proteins in LN-308 treated with Thapsigargin and Tunicamycin for 6, 16, 24 hr, the amount of spiked-in synthetic peptides varied from 29.5 amol (APLSPSLLIR, XBP1) to 490.4 amol (YLTDFEPIQCLGR, E2AK3) in roughly 1 µg of total peptide from samples. For the quantification of UPR protein in six glioblastoma cell lines, the absolute amount of tryptic peptides in each samples were determined using amino acid analysis approach (AAA). 1 µg of the tryptic peptides was used. 29.5 amol (APLSPSLLIR, XBP1) to 490.4 amol (YLTDFEPIQCLGR, E2AK3) of synthetic peptides were used for the quantification.

### Liquid chromatography and mass spectrometry

The peptide samples were separated on an Ultimate 3000 Rapid Separation Liquid chromatography (RSLC) nano system with ProFlow flow control device coupled to a Q Exactive HF mass spectrometer (both from Thermo Scientific, Hamburg, Germany). On the nano liquid chromatography (nanoLC) system, peptides were concentrated on a trapping-column (Acclaim C18 PepMap100, 100 µm 2 cm) using 0.1% TFA at a flowrate of 20 µl/min and subsequently separated on a reverse phase main-column (Acclaim C18 PepMap100, 75 µm 50 cm, Thermo Scientific, Waltham, MA, USA) using a binary gradient consisted of A: 0.1% formic acid (FA) and B: 84% acetonitrile, 0.1% FA at a flowrate of 250 nl/min. The gradient increased linearly from 3% A to 29.7% B over 75 min. Three washing steps at 95% of organic solvent B at the end of the gradient were applied to prevent carry-over into the next run. For the mass spectrometry (MS) analysis, the normalized collision energy was set at 27% and AGC target value at 3 × 10^6^. The resolution, isolation width and injection time were set as described in each paragraph. For the measurement with Orbitrap Fusion Lumos Tribrid Mass Spectrometer, the normalized collision energy was set at 32% and AGC target value at 3 × 10^6^. The resolution was set at 240,000, isolation width m/z value of 0.4 m/z and injection time at 500 ms.

### Data analysis using skyline and R

The raw files after measurement were imported to Skyline 64-bit version 3.7.0.11317^51^. All the acquired data were reviewed and integrated manually. For the calibration curves, both light and heavy peptides were only defined as detected if their mass deviation was less than 5 ppm, at least 2 transitions were detected, and the peaks could be observed in Gauss distribution form and differentiated from the background. Furthermore, the heavy and light peptides must have identical retention time and transition ratios. The lowest concentrations of the dilution series, which had less than 20% of deviation, were defined as limit of quantification for the particular peptide at certain parameter setting. After analysis in Skyline, the data was imported in.csv format into R version 3.4.3 (2017-11-30)–“Kite-Eating Tree”^52^ for the calculation and graphic illustrations. For the calculation and data formatting, packages *reshape2*^53^ were used and for the graphical illustrations *ggplot2*^54^, *gridExtra*^55^ and *scales*^56^ packages. The measurements were performed at least in three technical or biological replicates as described in each paragraph. Average values were determined using function *mean* of R and standard deviation using *sd*. For the absolute quantification of UPR proteins in LN308 cells non-treated and treated with Tm and Th for 24 hr using the optimized PRM method, the functions of the linear regression lines derived from the calibration curves using 240 k-500 ms parameter sets were used to calculate the absolute amount based on the MS response and the absolute amount of spiked-in internal standard. To calculate the copy number per cell, we determined the total protein amount per cell (0.20 ng +/− 0.03 ng) using BCA protein determination kit. A total peptide amount of 1 µg derived from the entire cell lysate complex was used for each measurement. First, the mol amount of each UPR peptides per cell was calculated and then multiplied with the Avogadro constant (6.022 × 10^23^ mol^-1^), which results in the copy number per cell for each UPR peptides. The scan frequencies of Lumos and Q Exactive HF raw files were calculated and plotted using *rawDiag* R package^57^. The R-scripts used for the calibration curve analysis, endogenous peptide level calculation and calculation of copy number per cell are supplied in the Supplementary Document.

## Supplementary information

Supplementary information

## Data Availability

The extracted ion chromatograms of all measurements can be found in this Skyline Panorama link (https://panoramaweb.org/signaling_targeted_proteomics_2018.url).
