# Supplementary material for: A sensitive and simple targeted proteomics approach to quantify transcription factor and membrane proteins of the unfolded protein response pathway in glioblastoma cells
Source: Sci Rep. 2019 Jun 20;9:8836. doi: 10.1038/s41598-019-45237-5 (PMC6586633; doi:10.1038/s41598-019-45237-5)
Supplement: Supplementary file 1 — Supplementary information [file 41598_2019_45237_MOESM1_ESM.pdf]

## Supplementary Information

# A sensitive and simple targeted proteomics approach to quantify transcription factor and membrane proteins of the unfolded protein response pathway in glioblastoma cells

Chi DL Nguyen[1], Sebastian Malchow[1], Stefan Reich[4], Sascha Steltgens[7], Konstantin V Shuvaev[1], Stefan Loroch[1], Christin Lorenz[1], Albert Sickmann[1,5,6], Christiane B. Knobbe-Thomsen[7], Björn Tews[2,3], Jan Medenbach[4], Robert Ahrends[1*]

1) Leibniz-Institut für Analytische Wissenschaften-ISAS-e.V., 44227 Dortmund, Germany
2) Schaller Research Group, University of Heidelberg and DKFZ, 69120 Heidelberg, Germany
3) Molecular Mechanisms of Tumor Invasion, DKFZ, 69120 Heidelberg, Germany
4) Translational Control Group, Biochemistry I, University of Regensburg, 93053 Regensburg, Germany
5) Medizinische Fakultät, Ruhr-Universität Bochum, Bochum 44801, Germany
6) College of Physical Sciences, University of Aberdeen, Old Aberdeen AB24 3UE, UK
7) Institute of Neuropathology, Medical Faculty, Heinrich-Heine-University Düsseldorf, 40225 Düsseldorf, Germany

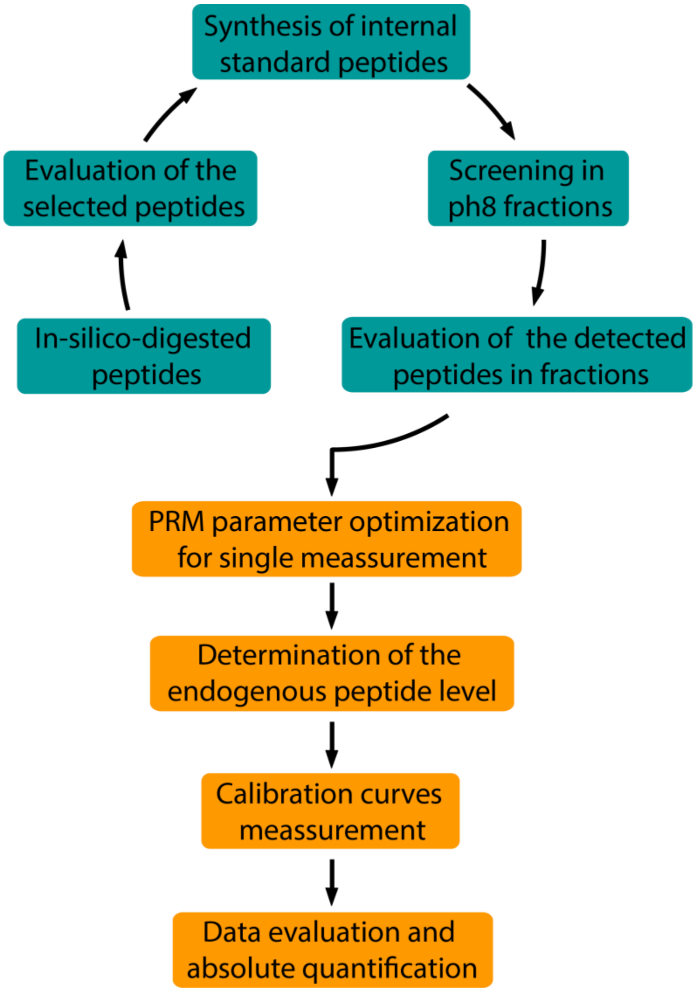


**Supplementary Figure S1. Workflow to establish a PRM assay for quantification of low abundancy proteins.** For low abundancy proteins, the proteotypic peptides can be selected from in-silico-digest database with trypsin as enzyme using Skyline version 3.7.0.11317 64-bit and Uniprot human database (downloaded on 22sd of July 2015). After evaluation of the peptides on fractionated samples, we synthesized the isotope coded internal standard of the target peptides and performed a standard PRM screening (60,000 resolution, 1.6m/z isolation window, 3e6 AGC target and 80ms injection time) on the ph8 fractionated samples. The detected peptides are further used in the PRM parameter optimization. Three major PRM parameters (resolution, injection time and isolation width) were optimized for a direct PRM measurement of the proteins/peptides of interest. After the PRM parameter optimization, the endogenous level of the proteins/peptides of interest was estimated based on the spiked in amount of IS. For the absolute quantification calibration curves were performed for each peptide.

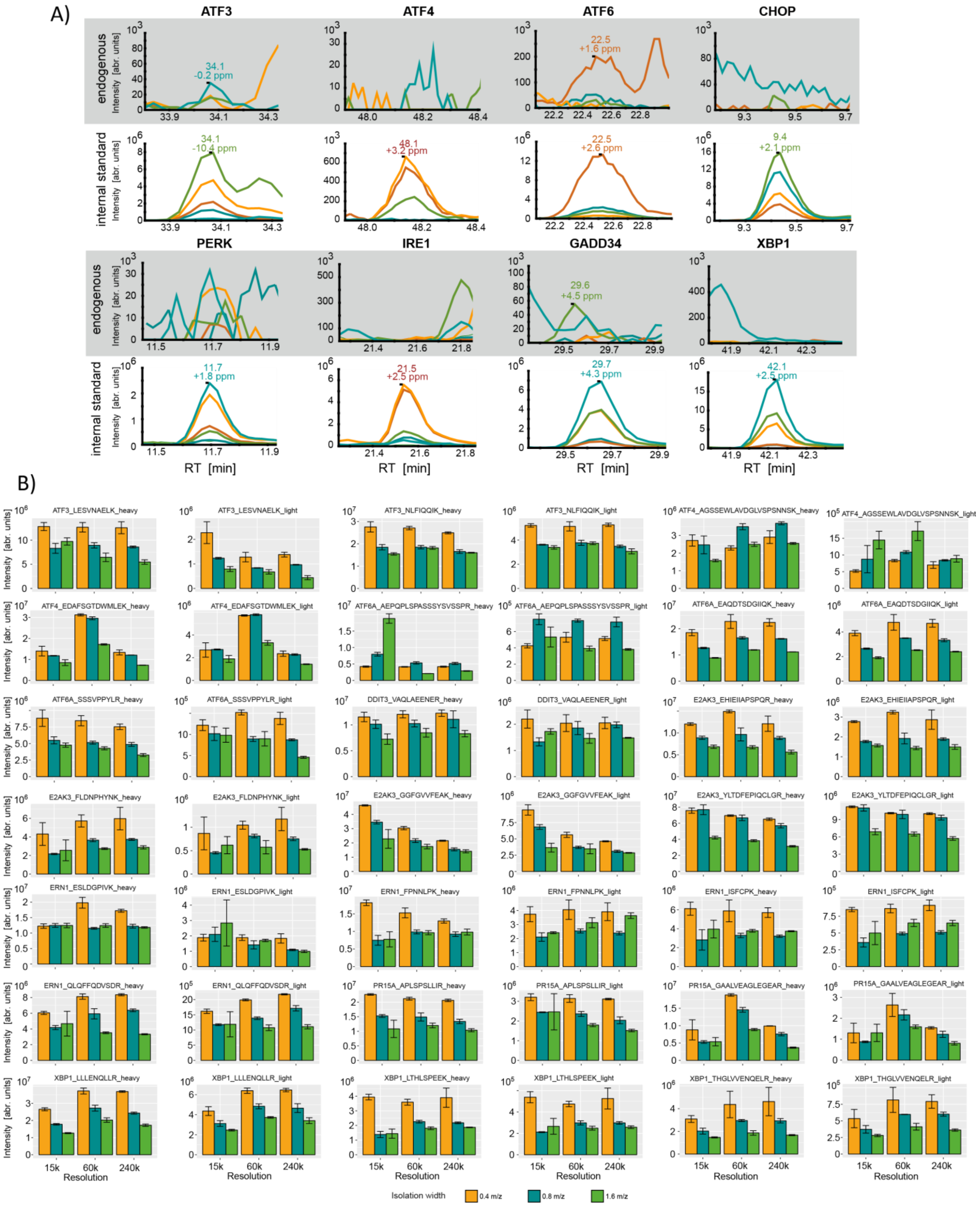


**Supplementary Figure S2. Initial UPR screen and the effect of different isolation widths on peak intensity.** A) PRM-XICs before assay optimization of representative UPR target peptides using standard PRM parameters (60,000 resolution, 80ms injection time, 1.6m/z isolation width, 3e6 AGC target value). The endogenous peptides are marked with gray background and the internal standard peptides with white. B) The isolation widths were set at 0.4, 0.8 and 1.6m/z and resolution at 15k, 60k and 240k respectively. The intensity is the sum of all fragment ion peak area. All the peptides could be detected with higher intensity at 0.4m/z compared to 0.8 and 1.6m/z. Three measurements were performed for each parameter set.

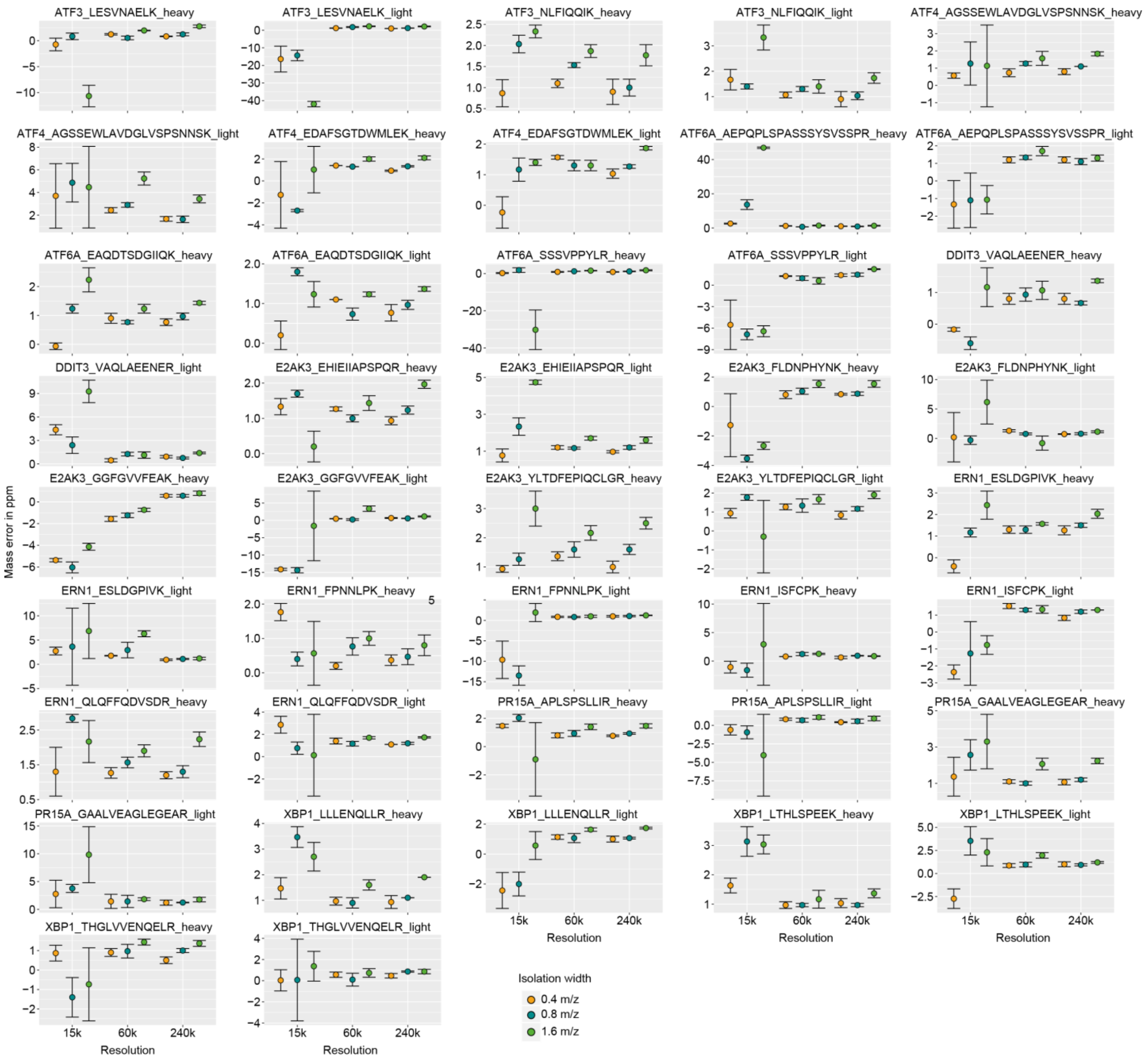


**Supplementary Figure S3. Effect of different isolation widths on the average mass error of the detected peak**. The isolation widths were set at 0.4, 0.8 and 1.6m/z and resolution at 15k, 60k and 240k respectively. The mass error in ppm is the average value of all transitions of a particular precursor. Three measurements were performed for each parameter set.

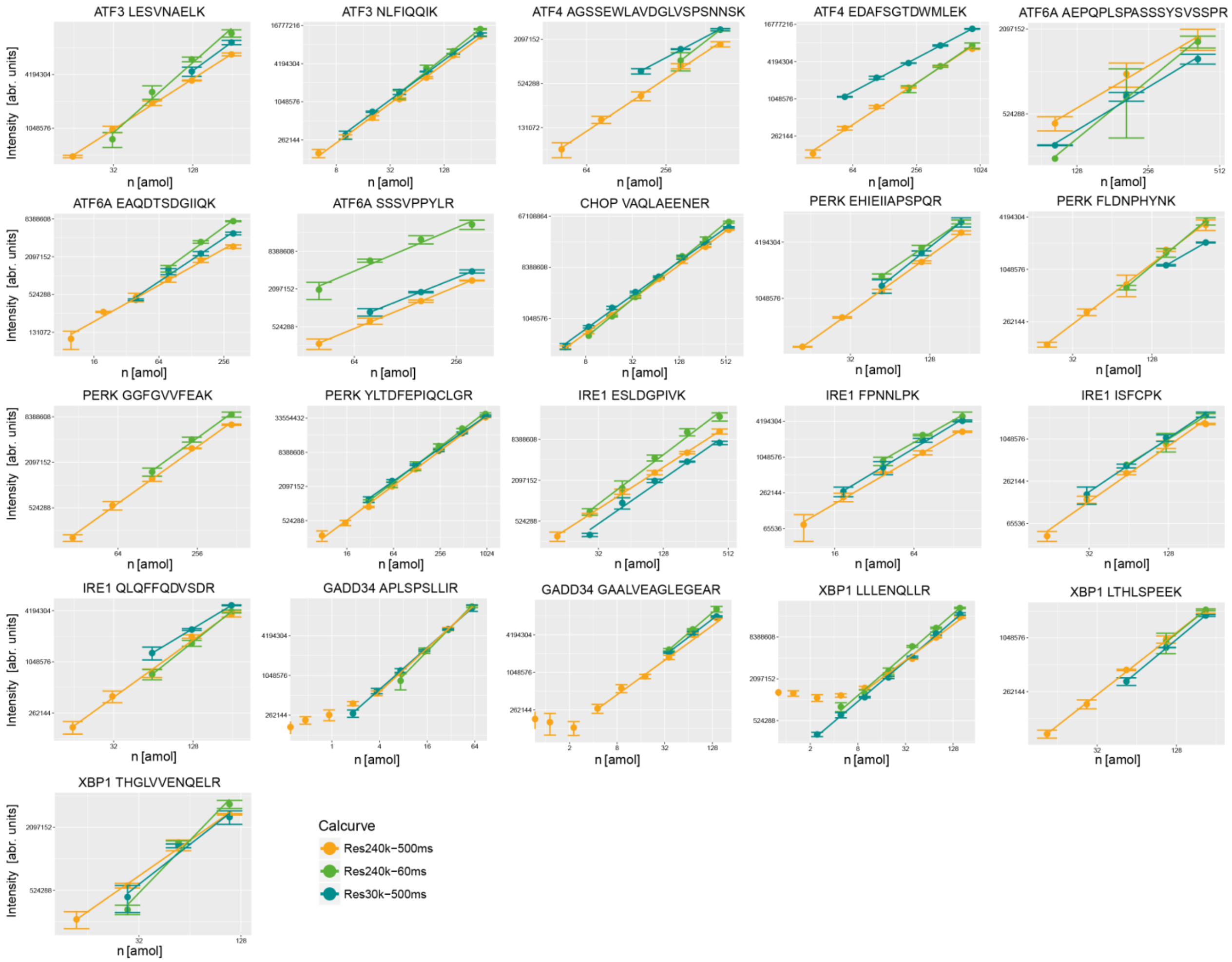


**Supplementary Figure S4. Calibration curves for all peptides using three different parameter set-ups.** The sensitivity of three different parameter set-ups: 240k resolution 500ms injection time, 240k-60ms and 30k-500ms were investigated on all 21 peptides. The axes are presented in log2 scale. Each calibration point was performed in three replicates.

| Protein Name | Peptide Sequence | Limit of Quantification [amol] | | |
|---|---|---|---|---|
| | | 240k-500ms | 240k-60ms | 30k-500ms |
| **ATF3 (1/2)** | **LESVNAELK** | 15.74 | 31.48 | 125.91 |
| **ATF3 (2/2)** | **NLFIQQIK** | 5.07 | 40.60 | 10.15 |
| **ATF4 (1/2)** | **AGSSEWLAVDGLVSPSNNSK** | 82.76 | 662.09 | 165.52 |
| **ATF4 (2/2)** | **EDAFSGTDWMLEK** | 26.91 | 215.24 | 53.81 |
| **ATF6A (1/3)** | **AEPQPLSPASSSYSVSSPR** | 103.19 | 412.77 | 103.19 |
| **ATF6A (2/3)** | **EAQDTSDGIIQK** | 19.58 | 78.33 | 39.17 |
| **ATF6A (3/3)** | **SSSVPPYLR** | 39.56 | 79.12 | 79.12 |
| **CHOP (1/1)** | **VAQLAEENER** | 4.39 | 17.54 | 4.39 |
| **PERK (1/4)** | **EHIEIIAPSPQR** | 13.86 | 55.44 | 110.87 |
| **PERK (2/4)** | **FLDNPHYNK** | 20.49 | 81.96 | 163.93 |
| **PERK (3/4)** | **GGFGVVFEAK** | 29.07 | 116.27 | N.D |
| **PERK (4/4)** | **YLTDFEPIQCLGR** | 15.32 | 30.65 | 30.65 |
| **IRE1 (1/4)** | **ESLDGPIVK** | 13.31 | 26.61 | 26.61 |
| **IRE1 (2/4)** | **FPNNLPK** | 18.63 | 37.26 | 18.63 |
| **IRE1 (3/4)** | **ISFCPK** | 15.35 | 245.61 | 61.40 |
| **IRE1 (4/4)** | **QLQFFQDVSDR** | 15.67 | 62.69 | 62.69 |
| **GADD34 (1/2)** | **APLSPSLLIR** | 3.69 | 14.75 | 1.84 |
| **GADD34 (2/2)** | **GAALVEAGLEGEAR** | 4.52 | 36.19 | 36.19 |
| **XBP1 (1/3)** | **LLLENQLLR** | 9.75 | 4.88 | 2.44 |
| **XBP1 (2/3)** | **LTHLSPEEK** | 13.35 | 213.63 | 53.41 |
| **XBP1 (3/3)** | **THGLVVENQELR** | 13.69 | 54.77 | 54.77 |

**Supplementary Table S1. Limit of quantification of all peptides at different parameter set-ups.** The LOQs of each peptide were determined for each parameter set-ups (240k-500ms, 240k-60ms, 30k-500ms). The LOQ is defined as the lowest amount on column with a standard deviation less than 20%.

| Protein Name | Peptide Sequence | Analyzed amount at each calibration point [amol] | | | | | | | | |
|---|---|---|---|---|---|---|---|---|---|---|
| | | Cal8 | Cal7 | Cal6 | Cal5 | Cal4 | Cal3 | Cal2 | Cal1 | Cal0 |
| ATF3 (1/2) | LESVNAELK | 251.83 | 125.91 | 62.96 | 31.48 | 15.74 | 7.87 | 3.93 | 1.97 | 0.00 |
| ATF3 (2/2) | NLFIQQIK | 324.77 | 162.38 | 81.19 | 40.60 | 20.30 | 10.15 | 5.07 | 2.54 | 0.00 |
| ATF4 (1/2) | AGSSEWLAVDGLVSPSNNSK | 662.09 | 331.05 | 165.52 | 82.76 | 41.38 | 20.69 | 10.35 | 5.17 | 0.00 |
| ATF4 (2/2) | EDAFSGTDWMLEK | 860.97 | 430.48 | 215.24 | 107.62 | 53.81 | 26.91 | 13.45 | 6.73 | 0.00 |
| ATF6A (1/3) | AEPQPLSPASSSYSVSSPR | 412.77 | 206.39 | 103.19 | 51.60 | 25.80 | 12.90 | 6.45 | 3.22 | 0.00 |
| ATF6A (2/3) | EAQDTSDGIIQK | 313.33 | 156.67 | 78.33 | 39.17 | 19.58 | 9.79 | 4.90 | 2.45 | 0.00 |
| ATF6A (3/3) | SSSVPPYLR | 316.47 | 158.23 | 79.12 | 39.56 | 19.78 | 9.89 | 4.94 | 2.47 | 0.00 |
| CHOP (1/1) | VAQLAEENER | 561.37 | 280.69 | 140.34 | 70.17 | 35.09 | 17.54 | 8.77 | 4.39 | 0.00 |
| PERK (1/4) | EHIEIIAPSPQR | 221.75 | 110.87 | 55.44 | 27.72 | 13.86 | 6.93 | 3.46 | 1.73 | 0.00 |
| PERK (2/4) | FLDNPHYNK | 327.85 | 163.93 | 81.96 | 40.98 | 20.49 | 10.25 | 5.12 | 2.56 | 0.00 |
| PERK (3/4) | GGFGVVFEAK | 465.09 | 232.55 | 116.27 | 58.14 | 29.07 | 14.53 | 7.27 | 3.63 | 0.00 |
| PERK (4/4) | YLTDFEPIQCLGR | 980.76 | 490.38 | 245.19 | 122.60 | 61.30 | 30.65 | 15.32 | 7.66 | 0.00 |
| IRE1 (1/4) | ESLDGPIVK | 425.79 | 212.90 | 106.45 | 53.22 | 26.61 | 13.31 | 6.65 | 3.33 | 0.00 |
| IRE1 (2/4) | FPNNLPK | 149.03 | 74.52 | 37.26 | 18.63 | 9.31 | 4.66 | 2.33 | 1.16 | 0.00 |
| IRE1 (3/4) | ISFCPK | 245.61 | 122.80 | 61.40 | 30.70 | 15.35 | 7.68 | 3.84 | 1.92 | 0.00 |
| IRE1 (4/4) | QLQFFQDVSDR | 250.75 | 125.38 | 62.69 | 31.34 | 15.67 | 7.84 | 3.92 | 1.96 | 0.00 |
| GADD34 (1/2) | APLSPSLLIR | 58.99 | 29.49 | 14.75 | 7.37 | 3.69 | 1.84 | 0.92 | 0.46 | 0.00 |
| GADD34 (2/2) | GAALVEAGLEGEAR | 144.75 | 72.38 | 36.19 | 18.09 | 9.05 | 4.52 | 2.26 | 1.13 | 0.00 |
| XBP1 (1/3) | LLLENQLLR | 156.03 | 78.02 | 39.01 | 19.50 | 9.75 | 4.88 | 2.44 | 1.22 | 0.00 |
| XBP1 (2/3) | LTHLSPEEK | 213.63 | 106.81 | 53.41 | 26.70 | 13.35 | 6.68 | 3.34 | 1.67 | 0.00 |
| XBP1 (3/3) | THGLVVENQELR | 109.53 | 54.77 | 27.38 | 13.69 | 6.85 | 3.42 | 1.71 | 0.86 | 0.00 |

**Supplementary Table S2. Amount on column of each peptide at each calibration point in amol.** From the highest amount (Cal8), the peptide mixture was diluted to half in matrix for the next calibration point. At Cal0, no peptide mixture was added in the matrix. Each calibration point was performed in three replicates.

| Protein Name | Peptide Sequence | DMSO | | Thapsigargin | | Tunicamycin | |
|---|---|---|---|---|---|---|---|
| | | c [copy/cell] | SD [copy/cell] | c [copy/cell] | SD [copy/cell] | c [copy/cell] | SD [copy/cell] |
| ATF3 (1/2) | LESVNAELK | 2944.00 | 449.88 | 27730.33 | 5205.11 | 6500.00 | 1050.09 |
| ATF3 (2/2) | NLFIQQIK | 5808.33 | 1223.40 | 44295.33 | 11269.73 | 13409.00 | 548.05 |
| ATF4 (1/2) | AGSSEWLAVDGLVSPSNNSK | 6571.33 | 1803.59 | 41448.33 | 4952.14 | 19236.00 | 7990.49 |
| ATF4 (2/2) | EDAFSGTDWMLEK | 4158.00 | 550.81 | 87390.00 | 10895.68 | 39152.33 | 17919.37 |
| ATF6A (1/3) | AEPQPLSPASSSYSVSSPR | 19166.67 | 651.72 | 34938.00 | 3570.22 | 11629.33 | 1826.07 |
| ATF6A (2/3) | EAQDTSDGIIQK | 61822.67 | 1724.88 | 116530.00 | 13107.15 | 38472.67 | 4650.64 |
| ATF6A (3/3) | SSSVPPYLR | 99546.67 | 11248.49 | 150610.67 | 20334.24 | 56993.33 | 5942.44 |
| CHOP (1/1) | VAQLAEENER | 677.33 | 168.97 | 51435.67 | 4087.90 | 21060.67 | 2204.45 |
| PERK (1/4) | EHIEIIAPSPQR | 12852.67 | 578.23 | 17225.00 | 3639.24 | 11662.67 | 1997.34 |
| PERK (2/4) | FLDNPHYNK | 9180.33 | 1102.62 | 20695.00 | 2948.96 | 10471.00 | 953.87 |
| PERK (3/4) | GGFGVVFEAK | 40200.33 | 3304.23 | 61853.33 | 7261.24 | 42075.00 | 3146.83 |
| PERK (4/4) | YLTDFEPIQCLGR | 22608.00 | 2012.15 | 48420.33 | 7177.81 | 24583.33 | 1984.16 |
| IRE1 (1/4) | ESLDGPIVK | 3169.67 | 82.71 | 14968.33 | 1922.77 | 7239.67 | 235.97 |
| IRE1 (2/4) | FPNNLPK | 6847.00 | 519.24 | 21565.67 | 1495.21 | 11100.33 | 423.19 |
| IRE1 (3/4) | ISFCPK | 7621.33 | 201.85 | 25047.67 | 323.53 | 12393.67 | 793.88 |
| IRE1 (4/4) | QLQFFQDVSDR | 7804.00 | 463.22 | 24934.67 | 1134.59 | 12841.67 | 1493.96 |
| GADD34 (1/2) | APLSPSLLIR | 234.33 | 41.68 | 6466.00 | 710.61 | 1396.00 | 303.35 |
| GADD34 (2/2) | GAALVEAGLEGEAR | 904.00 | 174.06 | 28244.33 | 4788.49 | 5481.00 | 2145.72 |
| XBP1 (1/3) | LLLENQLLR | 521.67 | 71.45 | 39887.00 | 6987.53 | 7619.67 | 1472.10 |
| XBP1 (2/3) | LTHLSPEEK | 297.33 | 175.48 | 23782.00 | 3842.87 | 5926.67 | 994.90 |
| XBP1 (3/3) | THGLVVENQELR | 442.00 | 20.81 | 18324.00 | 2561.39 | 4562.33 | 1357.72 |

**Supplementary Table S3. Copy number per cell of each UPR major signaling proteins in control treated cells (DMSO) and upon induction of ER stress (treated with Tunicamycin and Thapsigargin for 24 hours).** All the meassurements were performed in 3 biological replicates.

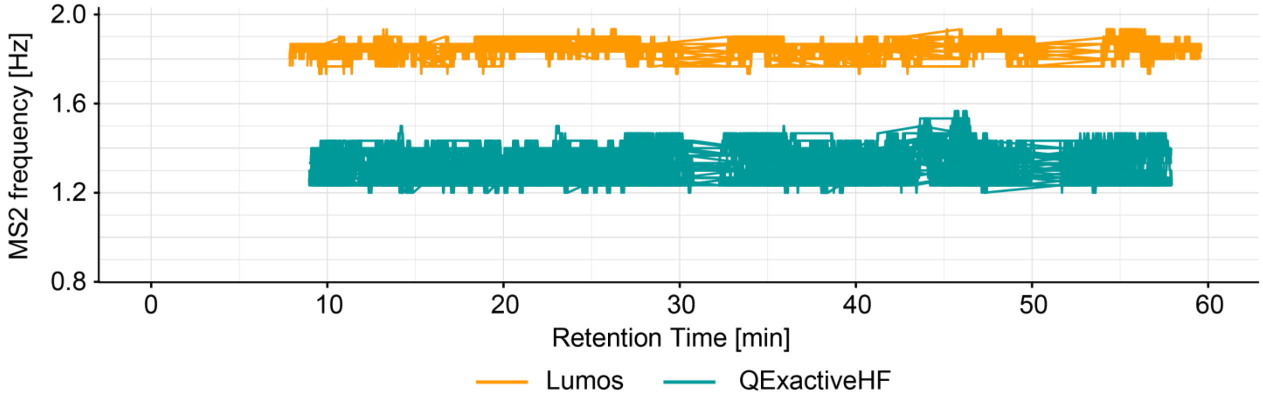


**Supplementary Figure S5. Comparison of scan frequency between Lumos and QExactiveHF Mass spectrometer**. Similar parameters were set on both instruments: 500ms injection time, 240,000 resolution, 0.4m/z isolation window. The scan frequency values of 54 Lumos raw files and 57 QExactiveHF raw files were calculated and illustrated.

```
########################################################################################
####################################
# R-script used for data analysis

########################################################################################
####################################
# calibration curve for 240k-500ms; 240k-60ms; 30k-500ms
########################################################################################
####################################

# reading export data file from skyline
mydirectory="H:/working documents/R-Analysis_tMSMS"
myproject="20180328_Calcurve_180307CN1"

calcurve1="20180618_CalCurve1_180307CN1.csv" # 240k-500ms
calcurve2="20180618_CalCurve2_180307CN1.csv" # 240k-60ms
calcurve3="20180618_CalCurve3_180307CN1.csv" # 30k-500ms

concentration="20180403_DilutionSerie_CalcurveUPR.txt" #calculated amount of calibration samples
[amol on column]

# export data file from skyline should contain the folowing columns (and example of the first row):
# "Protein Name"  "Peptide Sequence" "Isotope Label Type" "File Name" "Replicate Name" "Total Area
Fragment"
# sp|O75460|ERN1_HUMAN FPNNLPK light QExactiveHF02_06952.raw Cal0_1  3185164
cal1<-read.csv(file = paste(mydirectory, myproject, calcurve1, sep="/"),header = TRUE)
cal2<-read.csv(file = paste(mydirectory, myproject, calcurve2, sep="/"),header = TRUE)
cal3<-read.csv(file = paste(mydirectory, myproject, calcurve3, sep="/"),header = TRUE)
conc<-read.table(file = paste(mydirectory, myproject, concentration, sep="/"),header = TRUE, quote="")

# formating data after import
formating.input.data<-function(data,...){#begin of a function
  library(reshape2)
```

```
  #formating
  data$Total.Area.Fragment<-gsub("#N/A","0",data$Total.Area.Fragment)
  data$Total.Area.Fragment<-as.numeric(data$Total.Area.Fragment)
  newcolsname<-c("Name", "Technical.Replicate")
  newcolsname<- colsplit(as.character(data$Replicate.Name), "_", newcolsname)
  data<-cbind(data,newcolsname)
  return(data)
}#end of a function
calculate.mean.sd.calcurve<-function(data,...){#begin of a function
  #calculating mean and sd
  data$Replicate.Name<-NULL
  data1<-aggregate(Total.Area.Fragment ~ Protein.Name+Peptide.Sequence+Isotope.Label.Type+Name,
data=data, FUN=mean)
  data1$SD<-aggregate(Total.Area.Fragment ~
Protein.Name+Peptide.Sequence+Isotope.Label.Type+Name, data=data, FUN=sd)$Total.Area.Fragment
  return(data1)
}#end of a function
concentration.matching<-function(data,conc,curve,...){#begin of a function
  #matching with the concentration
  data$Peptide.Sequence<-paste(data$Peptide.Sequence,data$Name,sep="_")
  conc<-melt(conc)
  conc$Peptide<-paste(conc$Peptide,conc$variable, sep="_")
  data$Amount <- conc$value[match(data$Peptide.Sequence, conc$Peptide)]
  data$Peptide.Sequence<-gsub("_.*","",data$Peptide.Sequence)
  data$Identifier<-paste(gsub('.*\\|(.*)_.*','\\1',data$Protein.Name),data$Peptide.Sequence,sep="_")
  data$Name<-paste(curve,"_",data$Name,sep="")
  return(data)
}#end of a function
#   cal1 Res240k-500ms
formated_cal1<-formating.input.data(data=cal1)
calculated_cal1<-calculate.mean.sd.calcurve(data=formated_cal1)
calculated_cal1$SD_percent<-
as.numeric(calculated_cal1$SD/calculated_cal1$Total.Area.Fragment*100)
```

```
conc_cal1<-concentration.matching(data=calculated_cal1,conc = conc, curve="Res240k-500ms")
#   cal2 Res240k-60ms
formated_cal2<-formating.input.data(data=cal2)
calculated_cal2<-calculate.mean.sd.calcurve(data=formated_cal2)
calculated_cal2$SD_percent<-
as.numeric(calculated_cal2$SD/calculated_cal2$Total.Area.Fragment*100)
conc_cal2<-concentration.matching(data=calculated_cal2,conc = conc, curve="Res240k-60ms")
#   cal3 Res30k-500ms
formated_cal3<-formating.input.data(data=cal3)
calculated_cal3<-calculate.mean.sd.calcurve(data=formated_cal3)
calculated_cal3$SD_percent<-
as.numeric(calculated_cal3$SD/calculated_cal3$Total.Area.Fragment*100)
conc_cal3<-concentration.matching(data=calculated_cal3,conc = conc, curve="Res30k-500ms")
#   join all three calibration curves to one data table
joined<-rbind(conc_cal1,rbind(conc_cal2,conc_cal3))
#   plot analysis results
shortened<-
joined[which(joined$Isotope.Label.Type=="heavy"),c("Identifier","Name","Total.Area.Fragment","SD","A
mount","SD_percent")]
all<-split(shortened,f=shortened$Identifier)

linear.range.plot<-function(data,plot_name,...){ #begin of a function
  library(ggplot2)
  cam <- rgb(247/255, 164/255, 24/255, 1)
  xanh <- rgb(2/255, 140/255, 140/255, 1)
  xanhla <- rgb(88/255, 176/255, 55/255, 1)

  newcolsname<-c("Calcurve", "Cal.Replicate")
  newcolsname<- colsplit(as.character(data$Name), "_", newcolsname)
  data<-cbind(data,newcolsname)
  data<-data[which(data$Total.Area.Fragment!=0),]
```

```
  plot<-ggplot(data, aes(x=Amount, y=Total.Area.Fragment, color=Calcurve)) +
    geom_point(size=4)+
    geom_errorbar(aes(ymax = Total.Area.Fragment + SD, ymin= Total.Area.Fragment -
SD),width=0.5,size=1) +
    geom_smooth(method=lm,se=FALSE) +
    scale_color_manual(values=c("#F7A418FF","#58B037FF","#028C8CFF"))+
    xlab("Peptide amount on column[amol]") + ylab("Total Fragment Area") + ggtitle(plot_name)+
    scale_x_continuous(trans='log2')+ scale_y_continuous(trans='log2')+
    theme(axis.text = element_text(size = 10, colour= "black"),
          axis.title= element_text(size=10, colour="black"),
          plot.title = element_text(size=10, colour="black"))

  return(plot)
} #end of a function
#   ploting the result
all_plot<-list()
for (i in 1:length(all)){
  plot<-linear.range.plot(data=all[[i]],plot_name = all[[i]]$Identifier[[1]])
  all_plot[[i]]<-plot
}
library(gridExtra)
grid.arrange(grobs = all_plot, ncol =3 )

###########################################################################################
####################################
# calculate absolute amount of peptide in 24hr samples based on cal curve and the copy number per cell
###########################################################################################
####################################

mydirectory="H:/working documents/R-Analysis_tMSMS"
myproject="20180328_Calcurve_180307CN1"

calcurve1="20180618_CalCurve1_180307CN1.csv"
```

```
concentration="20180403_DilutionSerie_CalcurveUPR.txt"

sample24="20180618_LN308_Tm_Th_24_180307CN1.csv"
sample16="20180702_LN308_Tm_Th_16_180307CN1.csv"
sample6="20180702_LN308_Tm_Th_6_180307CN1.csv"

# data input preparation
# export data file from skyline should contain the folowing columns (and example of the first row):
# "Protein Name"  "Peptide Sequence" "Isotope Label Type" "File Name" "Replicate Name" "Total Area Fragment"
# sp|O75460|ERN1_HUMAN FPNNLPK light QExactiveHF02_06952.raw Cal0_1  3185164

cal1<-read.csv(file = paste(mydirectory, myproject, calcurve1, sep="/"),header = TRUE)
conc<-read.table(file = paste(mydirectory, myproject, concentration, sep="/"),header = TRUE, quote="")
endo24<-read.csv(file = paste(mydirectory, myproject, sample24, sep="/"),header = TRUE)
endo16<-read.csv(file = paste(mydirectory, myproject, sample16, sep="/"),header = TRUE)
endo6<-read.csv(file = paste(mydirectory, myproject, sample6, sep="/"),header = TRUE)

# formating data after import
formating.input.data<-function(data,...){#begin of a function
  library(reshape2)
  #formating
  data$Total.Area.Fragment<-gsub("#N/A","0",data$Total.Area.Fragment)
  data$Total.Area.Fragment<-as.numeric(data$Total.Area.Fragment)
  newcolsname<-c("Name", "Technical.Replicate")
  newcolsname<- colsplit(as.character(data$Replicate.Name), "_", newcolsname)
  data<-cbind(data,newcolsname)
  return(data)
}#end of a function
calculate.mean.sd.calcurve<-function(data,...){#begin of a function
  #calculating mean and sd
  data$Replicate.Name<-NULL
```

```
  data1<-aggregate(Total.Area.Fragment ~ Protein.Name+Peptide.Sequence+Isotope.Label.Type+Name,
data=data, FUN=mean)
  data1$SD<-aggregate(Total.Area.Fragment ~
Protein.Name+Peptide.Sequence+Isotope.Label.Type+Name, data=data, FUN=sd)$Total.Area.Fragment
  return(data1)
}#end of a function
concentration.matching<-function(data,conc,curve,...){#begin of a function
  #matching with the concentration
  data$Peptide.Sequence<-paste(data$Peptide.Sequence,data$Name,sep="_")
  conc<-melt(conc)
  conc$Peptide<-paste(conc$Peptide,conc$variable, sep="_")
  data$Amount <- conc$value[match(data$Peptide.Sequence, conc$Peptide)]
  data$Peptide.Sequence<-gsub("_.*","",data$Peptide.Sequence)
  data$Identifier<-paste(gsub('.*\\|(.*)_.*','\\1',data$Protein.Name),data$Peptide.Sequence,sep="_")
  data$Name<-paste(curve,"_",data$Name,sep="")
  return(data)
}#end of a function

formated_cal1<-formating.input.data(data=cal1)
calculated_cal1<-calculate.mean.sd.calcurve(data=formated_cal1)
conc_cal1<-concentration.matching(data=calculated_cal1,conc = conc, curve="Res240k-500ms")

# calculating conc of endogenous peptide
formated_endo24<-formating.input.data(data=endo24)
formated_endo24$Identifier<-
paste(gsub('.*\\|(.*)_.*','\\1',formated_endo24$Protein.Name),formated_endo24$Peptide.Sequence,se
p="_")
formated_endo24<-formated_endo24[,c("Identifier", "Isotope.Label.Type", "File.Name", "Name",
"Technical.Replicate", "Total.Area.Fragment")]
library(reshape2)
formated_endo24<-dcast(formated_endo24, Identifier + File.Name + Name + Technical.Replicate ~
Isotope.Label.Type, value.var = "Total.Area.Fragment")
```

```
# based on the calibration curve
lm.parameter.calcurve.linear<-function(data,...){#begin of a function

  data1<-
data[which(data$Isotope.Label.Type=="heavy"),c("Identifier","Name","Total.Area.Fragment","SD","Amo
unt")]
  all<-split(data1,f=data1$Identifier)
  all_calcurve_para<-data.frame(matrix(ncol = 4, nrow = length(unique(data1$Identifier))))
  colnames(all_calcurve_para) <- c("Identifier","r_square", "slope", "intercept")

  for (i in 1:length(all)){
    x<-log2(all[[i]][,"Amount"])
    x[is.infinite(x)]<- 0
    y<-log2(all[[i]][,"Total.Area.Fragment"])
    y[is.infinite(y)]<- 0
    fit<-lm(y~x)
    all_calcurve_para$Identifier[[i]]<-unique(all[[i]]$Identifier)
    all_calcurve_para$r_square[[i]]<-summary(fit)$r.squared
    all_calcurve_para$slope[[i]]<-coef(fit)[2]
    all_calcurve_para$intercept[[i]]<-coef(fit)[1]
  }
  return(all_calcurve_para)
}#end of a function

lm_calcurve1<-lm.parameter.calcurve.linear(data=conc_cal1)

cal.amount.curve<-vector()
for (i in 1:nrow(formated_endo24)){
  value1<-(log2(formated_endo24[i,"light"])-lm_calcurve1[which(formated_endo24[i,"Identifier"] ==
lm_calcurve1$Identifier),"intercept"])
  value2<-value1/lm_calcurve1[which(formated_endo24[i,"Identifier"] ==
lm_calcurve1$Identifier),"slope"]
  cal.amount.curve[[i]]<-round(2^value2,2)
```

```
}
formated_endo24$cal.amount.curve<-cal.amount.curve

# calculating mean, sd and plot
formated_endo24_ave_sd<-aggregate(cal.amount.curve ~ Identifier+Name, data=formated_endo24,
FUN=mean)
formated_endo24_ave_sd$SD_curve<-aggregate(cal.amount.curve ~ Identifier+Name,
data=formated_endo24, FUN=sd)$cal.amount.curve

# Calculating the copie number per cell
# from BCA protein determination kit, I can have the amoun tof protein per LN308 cell: 0.20 ng +/- 0.03
total protein
# for 24hr LN308: 1ug of total peptide was used per injection
# -> y amol of particular peptide per 1000ng of total peptide injected -> x amol/per cell=  (y*0.2)/1000
# x amol/cell *1e-18 mol *6.022e23 molecules = z molecules per cell
# conclusion: z molecules/cell = ((y*0.2)/1000)*1e-18*6.022e23
LN308_24hr<-formated_endo24[,c(1,3,4,7)]
LN308_24hr$Copies.per.cell<-round(((LN308_24hr$cal.amount.curve*0.2)/1000)*1E-18*6.022E23,0)
LN308_24hr_ave<-aggregate(Copies.per.cell ~ Identifier+Name, data=LN308_24hr, FUN=mean)
LN308_24hr_ave$SD<-aggregate(Copies.per.cell ~ Identifier+Name, data=LN308_24hr,
FUN=sd)$Copies.per.cell

##########################################################################################
#####################################
# calculate amount of endogenous peptide in 6, 16, 24 samples based on heavy spiked-in
##########################################################################################
#####################################

mydirectory="H:/working documents/R-Analysis_tMSMS"
myproject="20180328_Calcurve_180307CN1"

sample24="20180618_LN308_Tm_Th_24_180307CN1.csv"
sample16="20180702_LN308_Tm_Th_16_180307CN1.csv"
```

```
sample6="20180702_LN308_Tm_Th_6_180307CN1.csv"
concentration="20180403_DilutionSerie_CalcurveUPR.txt"


# data input preparation
# export data file from skyline should contain the folowing columns (and example of the first row):
# "Protein Name"  "Peptide Sequence" "Isotope Label Type" "File Name" "Replicate Name" "Total Area Fragment"
# sp|O75460|ERN1_HUMAN FPNNLPK light QExactiveHF02_06952.raw Cal0_1  3185164

endo24<-read.csv(file = paste(mydirectory, myproject, sample24, sep="/"),header = TRUE)
endo16<-read.csv(file = paste(mydirectory, myproject, sample16, sep="/"),header = TRUE)
endo6<-read.csv(file = paste(mydirectory, myproject, sample6, sep="/"),header = TRUE)
conc<-read.table(file = paste(mydirectory, myproject, concentration, sep="/"),header = TRUE, quote="")

formating.input.data<-function(data,...){#begin of a function
  library(reshape2)
  #formating
  data$Total.Area.Fragment<-gsub("#N/A","0",data$Total.Area.Fragment)
  data$Total.Area.Fragment<-as.numeric(data$Total.Area.Fragment)
  newcolsname<-c("Name", "Technical.Replicate")
  newcolsname<- colsplit(as.character(data$Replicate.Name), "_", newcolsname)
  data<-cbind(data,newcolsname)
  return(data)
}#end of a function

formated_endo16<-formating.input.data(data=endo16)
formated_endo16$Identifier<-
paste(gsub('.*\\|(.*)_.*','\\1',formated_endo16$Protein.Name),formated_endo16$Peptide.Sequence,sep="_")
formated_endo16<-formated_endo16[,c("Identifier", "Isotope.Label.Type", "File.Name", "Name",
"Technical.Replicate", "Total.Area.Fragment")]
```

```
formated_endo16<-dcast(formated_endo16, Identifier + File.Name + Name + Technical.Replicate ~
Isotope.Label.Type, value.var = "Total.Area.Fragment")

formated_endo6<-formating.input.data(data=endo6)
formated_endo6$Identifier<-
paste(gsub('.*\\|(.*)_.*','\\1',formated_endo6$Protein.Name),formated_endo6$Peptide.Sequence,sep=
"_")
formated_endo6<-formated_endo6[,c("Identifier", "Isotope.Label.Type", "File.Name", "Name",
"Technical.Replicate", "Total.Area.Fragment")]
formated_endo6<-dcast(formated_endo6, Identifier + File.Name + Name + Technical.Replicate ~
Isotope.Label.Type, value.var = "Total.Area.Fragment")

formated_endo24<-formating.input.data(data=endo24)
formated_endo24$Identifier<-
paste(gsub('.*\\|(.*)_.*','\\1',formated_endo24$Protein.Name),formated_endo24$Peptide.Sequence,se
p="_")
formated_endo24<-formated_endo24[,c("Identifier", "Isotope.Label.Type", "File.Name", "Name",
"Technical.Replicate", "Total.Area.Fragment")]
formated_endo24<-dcast(formated_endo24, Identifier + File.Name + Name + Technical.Replicate ~
Isotope.Label.Type, value.var = "Total.Area.Fragment")

# based on the heavy spiked in
# 16hr
cal.amount.heavy<-vector()
for (i in 1:nrow(formated_endo16)){
  value1<-(formated_endo16[i,"light"]) * (conc[which(gsub(".*_","",formated_endo16[i,"Identifier"]) ==
conc$Peptide),"Cal7"]) / (formated_endo16[i,"heavy"])
  cal.amount.heavy[[i]]<-round(value1,2)
}
formated_endo16$cal.amount.heavy<-cal.amount.heavy
formated_endo16_ave_sd<-aggregate(cal.amount.heavy ~ Identifier+Name, data=formated_endo16,
FUN=mean)
```

```
formated_endo16_ave_sd$SD_heavy<-aggregate(cal.amount.heavy ~ Identifier+Name,
data=formated_endo16, FUN=sd)$cal.amount.heavy
formated_endo16_ave_sd$Incubation<-"16"
formated_endo16_ave_sd[formated_endo16_ave_sd$Name=="Dm","Name"]<-"DMSO"
formated_control_ave_sd<-
formated_endo16_ave_sd[which(formated_endo16_ave_sd$Name=="control"),]
formated_endo16_ave_sd<-
formated_endo16_ave_sd[which(formated_endo16_ave_sd$Name!="control"),]

# 6hr
cal.amount.heavy<-vector()
for (i in 1:nrow(formated_endo6)){
  value1<-(formated_endo6[i,"light"]) * (conc[which(gsub(".*_","",formated_endo6[i,"Identifier"]) ==
conc$Peptide),"Cal7"]) / (formated_endo6[i,"heavy"])
  cal.amount.heavy[[i]]<-round(value1,2)
}
formated_endo6$cal.amount.heavy<-cal.amount.heavy
formated_endo6_ave_sd<-aggregate(cal.amount.heavy ~ Identifier+Name, data=formated_endo6,
FUN=mean)
formated_endo6_ave_sd$SD_heavy<-aggregate(cal.amount.heavy ~ Identifier+Name,
data=formated_endo6, FUN=sd)$cal.amount.heavy
formated_endo6_ave_sd$Incubation<-"6"
formated_endo6_ave_sd[formated_endo6_ave_sd$Name=="Dm","Name"]<-"DMSO"

# 24hr
cal.amount.heavy<-vector()
for (i in 1:nrow(formated_endo24)){
  value1<-(formated_endo24[i,"light"]) * (conc[which(gsub(".*_","",formated_endo24[i,"Identifier"]) ==
conc$Peptide),"Cal7"]) / (formated_endo24[i,"heavy"])
  cal.amount.heavy[[i]]<-round(value1,2)
}
formated_endo24$cal.amount.heavy<-cal.amount.heavy
```

```
formated_endo24_ave_sd$cal.amount.heavy<-aggregate(cal.amount.heavy ~ Identifier+Name,
data=formated_endo24, FUN=mean)$cal.amount.heavy
formated_endo24_ave_sd$SD_heavy<-aggregate(cal.amount.heavy ~ Identifier+Name,
data=formated_endo24, FUN=sd)$cal.amount.heavy
formated_endo24_ave_sd$Incubation<-"24"

# merge all for plotting
mergeddata<-rbind(formated_endo6_ave_sd,rbind(formated_endo16_ave_sd,
                                  formated_endo24_ave_sd[,c("Identifier", "Name", "cal.amount.heavy",
"SD_heavy", "Incubation")]))
```
